# Structure-Based Engineering for PTPσ-Targeted Therapeutic Development

**DOI:** 10.3390/ijms27198659

**Published:** 2026-09-28

**Authors:** Hyunjin Kim, Seong Eon Ryu

**Affiliations:** Department of Bioengineering, College of Engineering, Hanyang University, Seoul 04673, Republic of Korea; he3640@hanyang.ac.kr

**Keywords:** PTPσ, PTPRS, receptor protein tyrosine phosphatase, structure-based engineering, proteoglycan switch, neural regeneration, rheumatoid arthritis

## Abstract

Protein tyrosine phosphatase sigma (PTPσ) is a member of the leukocyte common antigen-related (LAR) family of receptor-type protein tyrosine phosphatases. Its architecture integrates an extracellular proteoglycan-sensing region, a single-pass transmembrane helix, and tandem intracellular phosphatase domains. In neural injury, inhibition of PTPσ signaling can relieve chondroitin sulfate proteoglycan (CSPG)-mediated restriction of axonal growth and plasticity. In rheumatoid arthritis (RA), by contrast, restoration of PTPσ activity in fibroblast-like synoviocytes can suppress pathogenic migration and invasion. Structural studies have established a proteoglycan-switch model in which glycosaminoglycan engagement of the N-terminal immunoglobulin-like domains and extracellular clustering regulate intracellular phosphatase activity. Recent work demonstrated that antibodies to the membrane-proximal fibronectin type III-like domain 9 (Fn9) can regulate PTPσ oligomerization and syndecan-4 association in rheumatoid arthritis. Structure-guided protein engineering has converted a poorly soluble PTPσ Ig1–Ig2 decoy into developable Fc-fusion variants with substantially improved solubility and expression yield while retaining glycosaminoglycan binding and cell-based activity. This review summarizes the structural basis of PTPσ regulation and evaluates therapeutic modalities and engineering strategies for advancing PTPσ-directed biologics toward clinical candidates.

## 1. Introduction

Protein tyrosine phosphorylation is a reversible signaling mechanism that controls receptor signaling, adhesion, cytoskeletal organization, cell migration, proliferation, differentiation, survival, and synaptic communication [1,2,3,4]. Protein tyrosine kinases install phosphate groups on tyrosine residues, whereas protein tyrosine phosphatases (PTPs) remove these groups through hydrolysis of the phosphotyrosyl ester bond. The catalytic reaction is mediated by a conserved active-site cysteine in the signature motif C(X)5R, which forms a transient cysteinyl–phosphate intermediate; an invariant arginine stabilizes the substrate phosphate group, and an aspartate-containing general-acid loop contributes to catalysis. By opposing or reshaping kinase-driven phosphorylation, PTPs can terminate, tune, spatially confine, or redirect signaling rather than merely acting as passive signal “off switches.”

The human phosphatome comprises multiple structurally related PTP subfamilies whose biological functions extend beyond simple signal termination to spatial and temporal control of phosphorylation networks; this same active-site conservation has historically complicated the development of selective catalytic inhibitors [1,2,3,4,5,6,7,8,9]. Receptor-type PTPs couple this catalytic machinery to extracellular recognition. In PTPσ, the membrane-proximal cytoplasmic D1 domain is the principal catalytically competent phosphatase domain, whereas the distal D2 domain is a catalytically impaired pseudo-phosphatase that contributes to receptor regulation, localization, and/or intramolecular organization. Its extracellular domain recognizes proteoglycan-associated glycosaminoglycans (GAGs) and undergoes ligand-dependent changes in receptor association. Thus, extracellular glycan composition and multivalent presentation can regulate the accessibility, local concentration, and signaling state of the intracellular PTPσ catalytic machinery.

Protein tyrosine phosphatase sigma (PTPσ) is encoded by PTPRS and belongs to the type IIA receptor protein tyrosine phosphatase (RPTP) family, together with LAR and PTPδ [5,8,10,11,12]. RPTPs are attractive drug targets because their extracellular domains (ECDs) regulate intracellular phosphatase signaling, providing opportunities to control enzyme activity without directly engaging the highly conserved catalytic pocket [5,6,13]. In central nervous system injury, PTPσ functions as a receptor for neural regeneration-inhibitory chondroitin sulfate proteoglycans (CSPGs) enriched in glial scars and perineuronal nets [14,15,16,17,18,19], where the PTPσ activity is turned on by CSPGs. In addition to its role in injury responses, PTPσ belongs to a broader LAR-RPTP adhesion-signaling network that organizes synapse formation through trans-synaptic interactions and developmental axon-targeting programs [11,20,21,22,23,24,25,26]. Therapeutic inhibition of the PTPσ enzyme activity or disruption of CSPG–PTPσ signaling can therefore favor neurite extension, axonal sprouting, and circuit remodeling [14,15,16,27,28,29]. In rheumatoid arthritis, PTPσ activity is suppressed by interactions with heparan sulfate proteoglycans (HSPGs), including syndecan-4, in fibroblast-like synoviocytes (FLSs). Releasing this suppression restores PTPσ activity and attenuates invasive FLS behavior [30,31].

The recent availability of mechanistic structural information [22,32] and structure-guided engineering data [33] has transformed PTPσ from a biologically interesting receptor into a tractable biologics-design problem. The main question is no longer simply whether PTPσ can be targeted, but which domain, epitope, molecular format, valency, and exposure profile best produce the intended receptor regulation in a specific disease setting. Structural information on the PTPσ ectodomain has been leveraged to engineer Ig1–Ig2 decoy variants with improved solubility, expression yield, and overall developability while preserving glycosaminoglycan-binding activity [33]. In parallel, the membrane-proximal fibronectin type III-like domain 9 (Fn9)-directed antibodies and soluble Fn9-based decoys have revealed an additional extracellular strategy for regulating PTPσ by altering receptor association and oligomeric state [31]. Potential therapeutic applications of PTPσ modulation span neural injury and repair, synaptic and neurodegenerative disorders, rheumatoid arthritis, and hematopoietic regeneration; however, the desired direction of PTPσ modulation differs substantially across these settings [34,35,36]. In this review, we will discuss recently emerging therapeutic modalities based on structure-guided engineering and optimization.

PTPσ was selected for this review because it represents an unusually instructive receptor–phosphatase drug-discovery problem (Table 1). In contrast to cytosolic PTPs, whose catalytic sites must be reached inside cells, PTPσ offers extracellular epitopes that can be addressed by antibodies, soluble decoys, and Fc-fusion proteins. In contrast to many receptors regulated primarily by a single protein ligand, PTPσ integrates information from the identity, sulfation pattern, density, and spatial presentation of extracellular glycosaminoglycans. Moreover, the same receptor can have opposite desirable therapeutic directions across disease contexts: attenuation of PTPσ-dependent inhibitory signaling may be beneficial in neural injury and hematopoietic regeneration, whereas restoration of PTPσ activity may be beneficial in pathogenic synoviocytes in rheumatoid arthritis [17,19,30,32,34,35,36]. This context dependence makes PTPσ a valuable model for receptor-state engineering and highlights why affinity alone is insufficient for therapeutic candidate selection.

## 2. Structural and Mechanistic Basis for Therapeutic Engineering

### 2.1. Domain Organization

The PTPσ ectodomain contains three N-terminal immunoglobulin-like domains followed by nine fibronectin (Fn) type III-like domains (Figure 1) [5,10,12]. The N-terminal Ig1–Ig2 tandem adopts an elongated immunoglobulin-superfamily architecture and presents a continuous basic surface that binds sulfated glycosaminoglycans. Ig3 and the Fn repeats are expected to adopt β-sandwich immunoglobulin-superfamily folds, with individual FnIII modules contributing to ectodomain length, flexibility, spacing, and partner interactions. The membrane-proximal Fn9 module is of particular interest because antibody binding to this region can alter PTPσ association with syndecan-4 and receptor oligomerization [31]. The single-pass TM helix anchors the receptor and transmits extracellular organization to the cytoplasmic segment. The D1 and D2 domains have the classical PTP fold, consisting of a central β-sheet flanked by α-helices and containing the conserved PTP-loop active-site motif. D1 is catalytically active, whereas D2 is a regulatory pseudo-phosphatase with greatly reduced catalytic competence.

The N-terminal Ig1–Ig2 segment is the best-characterized ligand-recognition module. The structure of PTPσ Ig1–Ig2 identifies a positively charged, elongated glycosaminoglycan-binding surface [17,32,37]. This site supports interactions with sulfated glycan chains and provides a physical basis for recognition of both HSPGs and CSPGs [17,22]. Interestingly, the interaction’s glycosaminoglycan identity and multivalency influence receptor clustering and downstream enzymatic state [17,19,30,32,38]. High-resolution structural information is available for discrete PTPσ regions, including the N-terminal Ig1–Ig2 proteoglycan-recognition module, a six-domain N-terminal ectodomain fragment, and the intracellular D1–D2 phosphatase tandem (Table 2) [17,32,37,39,40]. However, an atomic-resolution structure of full-length, membrane-embedded PTPσ, including all nine fibronectin type III-like domains and ligand-dependent oligomeric states, has not yet been reported.

### 2.2. The Proteoglycan Switch

Both heparan sulfate (HS) and chondroitin sulfate (CS) are sulfated glycosaminoglycans composed of repeating disaccharide units, but their sugar composition and sulfation patterns differ [10,17,19,32]. HSPG-associated HS can engage and cluster multiple PTPσ molecules, which is associated with reduced PTPσ phosphatase activity; CSPG-associated CS can favor distinct receptor regulation associated with active PTPσ signaling in neural contexts (Figure 2) [17,19,30,32,38]. More broadly, the inhibitory effect of CSPG-rich extracellular matrices after CNS injury is mediated by several partially overlapping receptor systems, including LAR-family phosphatases and Nogo-receptor family members, and may be counteracted by strategies that modify the matrix or enhance integrin-dependent growth signaling [41,42,43,44,45,46,47]. The proteoglycan-dependent switch is thought to arise from differences in glycosaminoglycan composition, sulfation pattern, chain architecture, and multivalent presentation, which can impose distinct receptor-clustering geometries [17,32,38].

In experimental systems, multivalent HSPG engagement promotes clustering of PTPσ and related type IIA RPTPs and is associated with reduced phosphatase activity, whereas CSPG-associated ligands can support distinct signaling states in neural contexts [14,17,30,31,32]. Although the full three-dimensional structure of intact, membrane-embedded PTPσ in distinct ligand-bound states has not been resolved, the available biochemical and cellular data establish that extracellular ligand engagement communicates across the membrane to influence intracellular phosphatase function [14,31,32,38].

### 2.3. Intracellular Regulatory Features

The tandem D1–D2 cytoplasmic region offers a second therapeutic entry point. Catalytic-site inhibition is conceptually direct but carries the familiar selectivity challenge of the conserved PTP active site and requires cytosolic access [6,13]. PTP catalytic cysteines are redox sensitive [37]; oxidant-mediated regulation of receptor PTPs further highlights the chemical reactivity and context dependence of this target class [6]. ISP is a cell-penetrating peptide derived from the PTPσ juxtamembrane wedge region and has been used as a tool to attenuate PTPσ-dependent inhibitory signaling in preclinical injury models [27,28]. ISP binds PTPσ and produces functional effects consistent with releasing CSPG-mediated inhibition in neural injury models [27,28]. While this validates intracellular modulation, the modality has practical development challenges: cell penetration, biodistribution, proteolytic stability, exposure–response control, off-target engagement among related LAR-family proteins, and scalable peptide manufacturing/formulation [27,28].

## 3. Therapeutic Modalities and Structure-Based Opportunities

### 3.1. Soluble Ig1–Ig2 Decoy Proteins

Soluble PTPσ Ig1–Ig2 proteins act as extracellular decoys for proteoglycan ligands. This strategy can be particularly appropriate when disease pathology is driven by ligand-dependent PTPσ activation, such as CSPG-rich neural injury environments (Table 3) [14,33]. It can also be used to disrupt HSPG-mediated receptor regulation, depending on the biological context and construct mechanism (Table 3) [30,35]. The key advantage is topological simplicity: a decoy acts in the extracellular space and avoids the permeability and active-site selectivity constraints faced by intracellular target inhibitors. Fc fusion of the Ig1–Ig2 domain can extend serum half-life, enable Protein A platform purification, and support high avidity by dimeric presentation.

However, the primary limitation of the Ig1–Ig2 decoys has been the poor solubility of the native Ig1–Ig2 fragment [33]. A structure-based study directly addressed this developability problem (Figure 3). The study selected solvent-exposed hydrophobic residues in PTPσ Ig1–Ig2 that were spatially separated from the chondroitin sulfate interaction region [33]. Substitution of selected residues with alanine or asparagine generated variants with enhanced solubility and production yield (Table 4). The L155N/L187N variant maintained CS-binding and purification stability comparable to wild type, remained soluble at concentrations where wild-type protein precipitated, and showed enhanced functional performance in a SH-SY5Y neurite-outgrowth assay [33]. The L155N/L187N variant produced approximately 8.8-fold relative to wild type, whereas selected variants achieved approximately 5-fold improvement in solubility [33]. This is an instructive example of structure-based developability engineering that preserved the intended ligand-binding function.

### 3.2. Fn9-Targeting Antibodies and Domain Decoys

The characterization of anti-PTPσ antibodies provided a conceptual expansion of the targetable epitope landscape in PTPσ (Table 3) [31]. Antibodies 13G5, 22H8, and 49F2 were mapped to a shared or overlapping region in the membrane-proximal Fn9 domain (Figure 4) [31]. Notably, similar apparent epitope targeting and even favorable in vitro affinity did not guarantee equivalent cellular pharmacology, indicating an importance of geometrical states in the antibody-bound PTPσ ectodomain [31]. The 13G5 antibody reduced PTPσ association with syndecan-4 and reduced PTPσ oligomerization, thereby increasing cellular PTPσ activity and reducing FLS migration. The same study showed that isotype engineering could improve binding and cellular potency: switching an IgG1 constant region improved performance, while scFv-Fc formats also retained functional activity [31]. Here, isotype engineering refers to deliberate replacement or modification of an antibody-constant region (for example, conversion from an IgG2a- to an IgG1-based format) to alter Fc-mediated properties, hinge flexibility, valency-dependent geometry, half-life, effector function, and ultimately receptor clustering and cellular pharmacology without changing the antigen-binding variable domains.

This domain 9 can be exploited through more than one format: full IgG, scFv-Fc, or soluble Fn9-Fc decoy [31]. Soluble Fn9-Fc also reduced PTPσ–syndecan-4 proximity, PTPσ oligomerization, and FLS migration in a PTPσ-dependent manner. In an arthritis model, an engineered 13G5 IgG1 decreased clinical and histological measures of disease severity [31]. Although Fn9 has emerged as a functionally actionable membrane-proximal ectodomain epitope, an atomic-resolution structure of PTPσ Fn9 in complex with function-modulating antibodies or syndecan-4 has not yet been reported; accordingly, the structural basis of receptor-state modulation remains to be resolved.

### 3.3. Intracellular Peptides

ISP validates a structurally informed intracellular modulation approach [27,28]. A TAT-conjugated wedge-domain peptide was reported to bind PTPσ, cross biological barriers in preclinical studies, and promote axonal crossing of CSPG gradients (Table 3) [27]. In rodent spinal cord injury studies, systemic ISP delivery was associated with enhanced serotonergic innervation and improvement in locomotor and urinary outcomes [27,28]. For a therapeutic-development program, ISP-like molecules should be viewed as proof of mechanism and as a starting point for medicinal peptide optimization rather than a fully mature platform. Key engineering goals would include increased protease resistance, reduced nonspecific cell-penetrating-peptide effects, improved LAR-family selectivity, and defined exposure in the CNS or injury compartment.

ISP-like peptides could be advanced through a combined medicinal-chemistry and delivery strategy. Protease-sensitive positions may be replaced with D-amino acids, β-amino acids, N-methylated residues, or peptoid-like elements, while terminal capping, cyclization, stapling, or retro-inverso design may further increase proteolytic stability and conformational control [48]. Because cell-penetrating peptide-mediated uptake frequently results in endosomal sequestration, cytosolic delivery should be optimized using pH-responsive or fusogenic endosomal-escape motifs [49]. Liposomal, lipid-nanoparticle, polymeric-nanoparticle, or local depot formulations could additionally protect ISP from extracellular degradation and provide controlled CNS or lesion-site exposure [50].

### 3.4. Small Molecules and Allosteric Inhibitors

Small-molecule PTPσ inhibitors have been reported in preclinical settings, including compounds associated with hematopoietic stem-cell regeneration after radiation or chemotherapy-related bone-marrow injury [36]. The compound DJ001 is described as a selective, noncompetitive allosteric inhibitor of PTPσ (Table 3) [36]. Its advantages include potentially systemic and conventional drug-like administration, but intracellular access and selectivity optimization remain major risks. Active-site inhibitors of PTPs must overcome the high conservation and polar character of PTP catalytic pockets [6,13,34]. Allosteric strategies may provide improved selectivity, especially if they exploit PTPσ-specific interdomain interfaces, redox-responsive conformations, or juxtamembrane regulatory pockets. A structure-based discovery campaign could combine experimentally determined D1–D2 structures with fragment screening and molecular-dynamics-guided identification of cryptic pockets.

## 4. Disease Context

PTPσ participates in several biological systems, but the therapeutic objective is not uniform across them (Table 5). In neural injury, CSPG-rich extracellular matrices support PTPσ-dependent growth-inhibitory signaling, favoring interventions that interrupt ligand–receptor engagement or attenuate downstream PTPσ signaling. In rheumatoid arthritis, heparan sulfate proteoglycan-dependent organization of PTPσ in fibroblast-like synoviocytes suppresses its activity, favoring extracellular interventions that restore PTPσ function and restrain pathogenic motility. In hematopoietic stress, inhibition of PTPσ has been associated with enhanced hematopoietic stem-cell regeneration in preclinical studies. This disease-specific receptor-state logic provides the organizing principle for evaluating PTPσ therapeutic modalities.

### 4.1. Neural Injury and Neuroregeneration

Following central nervous system (CNS) injury, chondroitin sulfate proteoglycans (CSPGs) accumulate within the glial scar and extracellular matrix, where they form a major biochemical barrier to axonal extension and circuit reconstruction [14,15,18]. PTPσ is a key neuronal receptor for CSPG-associated glycosaminoglycan chains. Through its extracellular domains, PTPσ recognizes sulfated glycan motifs and transduces signals that promote growth-cone arrest, dystrophic end-bulb formation, and failure of axons to traverse CSPG-rich injured tissue [14,15,16,17]. CSPG-mediated regeneration failure is not determined solely by one receptor pathway. Matrix digestion by chondroitinase ABC, relief of NgR-family signaling, and restoration of integrin signaling have each promoted axonal growth or functional repair in experimental systems, supporting the rationale for combination approaches in which PTPσ modulation is paired with matrix-, growth-, or rehabilitation-based interventions [17,43,44,45,46,47]. This biology makes PTPσ an attractive intervention point because modulating the receptor could reduce inhibitory signaling without necessarily requiring complete removal of the extracellular scar, which also has structurally protective roles after injury (Table 5) [14,15,16,17].

Supporting this rationale, pharmacological modulation of PTPσ using intracellular sigma peptide (ISP) in spinal cord injury models improved multiple functional outcomes and increased serotonergic fiber innervation caudal to the lesion, consistent with enhanced neural connectivity or remodeling below the site of injury [27,28]. Nevertheless, interpretation of such findings requires careful experimental design, since increased innervation or behavioral recovery may reflect a combination of true long-distance axonal regeneration, collateral sprouting of spared pathways, synaptic reorganization, and activity-dependent plasticity. Therapeutic approaches for neural indications can be grouped into several complementary modalities: (i) cell-penetrating inhibitory peptides, intracellular biologics, or small molecules designed to attenuate PTPσ phosphatase activity; (ii) soluble extracellular-domain decoys, including Ig1–Ig2-based PTPσ fragments, that bind CSPG glycan ligands and thereby reduce their interaction with endogenous neuronal PTPσ; and (iii) engineered delivery formats that couple PTPσ-modulating payloads to brain-shuttle platforms or other CNS-targeting technologies [27,51].

### 4.2. Rheumatoid Arthritis

In rheumatoid arthritis (RA), fibroblast-like synoviocytes (FLSs) undergo a pathological phenotypic transition characterized by enhanced migration, invasion, resistance to apoptosis, and production of inflammatory mediators and matrix-degrading enzymes [52,53,54,55]. These activated FLS behave in part like transformed mesenchymal cells, spreading from the synovial lining into adjacent cartilage and bone and contributing directly to progressive joint destruction through secretion of cytokines, chemokines, matrix metalloproteinases, and osteoclast-supporting factors [52,53,54,55]. PTPσ signaling is an attractive extracellular regulatory axis in this context because its function in FLS can be modulated through HSPG-dependent interactions, including those involving syndecan-4 (Table 5) [10,30,31]. Because rheumatoid arthritis reflects both immune-mediated inflammation and tissue-intrinsic fibroblast persistence, a PTPσ-directed FLS therapy may be particularly valuable as a nonredundant complement to cytokine blockade rather than as a direct replacement for systemic immunomodulation [35,52,53,54,55,56].

Cell-based studies have suggested that decoy proteins containing the first and second immunoglobulin-like domains of PTPσ (Ig1–Ig2) and antibodies directed against the fibronectin type III repeat 9 (Fn9) region can interfere with PTPσ ligand interactions and/or receptor oligomerization, thereby suppressing pathogenic FLS motility [13,30,31]. In addition, an engineered 13G5 IgG1 antibody has been reported to reduce clinical arthritis severity and histologic evidence of joint damage in a mouse arthritis model, supporting the concept that extracellular targeting of the PTPσ pathway may attenuate disease-relevant synovial behavior [31].

RA is particularly suitable for development of an extracellular PTPσ-directed biologic because therapeutic activity does not require penetration of the blood–brain barrier, and both systemic administration and local intra-articular delivery could be considered depending on the intended disease population, joint distribution, pharmacokinetic profile, and safety margin. The program can also be supported by a practical translational assay package that directly links target engagement to functional outcomes: PTPσ–syndecan-4 proximity assays, measurements of receptor clustering or oligomerization, quantitative FLS migration and invasion assays, and analysis of downstream cytoskeletal pathways such as focal-adhesion remodeling, actin reorganization, and Rho-family GTPase-associated signaling [13,30,31].

### 4.3. Hematopoietic Regeneration

PTPσ has emerged as a negative regulator of hematopoietic stem cell (HSC) function and regenerative capacity [36,57]. Genetic studies showed that loss of PTPσ enhances long-term HSC repopulating activity, while PTPσ-negative human cord-blood HSCs exhibit substantially improved engraftment potential compared with PTPσ-positive cells (Table 5) [36]. Building on this mechanism, the small-molecule inhibitor DJ001 was developed as a selective, noncompetitive allosteric inhibitor of PTPσ [36]. In preclinical models, PTPσ inhibition promoted recovery of the HSC compartment after myeloablative stress, including ionizing radiation and 5-fluorouracil-induced chemotherapy. Systemic treatment of irradiated mice accelerated hematologic reconstitution and improved survival [36].

Mechanistically, PTPσ inhibition appears to protect and restore HSC function through activation of the Rho GTPase RAC1 pathway and increased expression of the anti-apoptotic protein BCL-XL, thereby reducing radiation-induced HSC apoptosis [36]. Importantly, treatment of irradiated human HSCs with DJ001 enhanced the recovery of cells capable of multilineage repopulation after transplantation into NSG mice, suggesting preservation of functional long-term repopulating HSCs rather than only short-term progenitors [36]. These findings position PTPσ inhibitors as a potential supportive therapeutic strategy for patients undergoing myelosuppressive chemotherapy, radiotherapy, or hematopoietic stem-cell transplantation.

## 5. Conclusions

PTPσ is a compelling example of a receptor whose therapeutic tractability emerges from understanding conformational and oligomeric regulation rather than from pursuing catalytic inhibition alone. The Ig1–Ig2 domain is a validated glycosaminoglycan-binding module suitable for decoy design, while Fn9 has emerged as an unexpected and therapeutically actionable regulatory site. Structure-based substitution of nonfunctional exposed hydrophobic residues has already demonstrated that PTPσ decoys can be made materially more soluble and productive without loss of ligand binding or cell-based activity [33]. In parallel, Fn9-directed antibodies and soluble Fn9 decoys demonstrate that extracellular control of receptor clustering can restore PTPσ activity and reduce pathogenic FLS behavior in RA models [31].

The central development principle is disease-specific receptor-state engineering. Neural regeneration programs generally seek to reduce PTPσ-mediated inhibitory signaling, whereas FLS-driven arthritis programs seek to restore PTPσ activity. Success will depend on choosing the appropriate mechanism, designing molecular geometry deliberately, and linking exposure to robust mechanistic pharmacodynamic biomarkers. With these principles, PTPσ-directed proteins, antibodies, peptides, and allosteric small molecules can progress toward promising therapeutic candidates.

To our knowledge, the PTPσ-modulating peptide NVG-291, a clinical-stage analog derived from the intracellular sigma peptide (ISP) approach, has entered publicly registered interventional clinical trials, including Phase 1 evaluation in healthy volunteers and a Phase 1b/2a trial in individuals with chronic cervical spinal cord injury. NVG-291 is designed to modulate PTPσ-mediated inhibitory signaling associated with chondroitin sulfate proteoglycans and thereby promote neural repair. Nevertheless, the broader PTPσ therapeutic field remains predominantly preclinical, encompassing extracellular Ig1–Ig2 decoys, Fn9-directed antibodies and soluble Fn9 decoys, ISP-based approaches, and small-molecule or allosteric inhibitors. Further clinical translation and development will require rigorous confirmation of target selectivity, receptor-state pharmacology, pharmacokinetics and tissue exposure, toxicology, and indication-specific pharmacodynamic biomarkers, as well as demonstration of clinically meaningful functional benefit in adequately powered controlled trials.

## Figures and Tables

**Figure 1:**
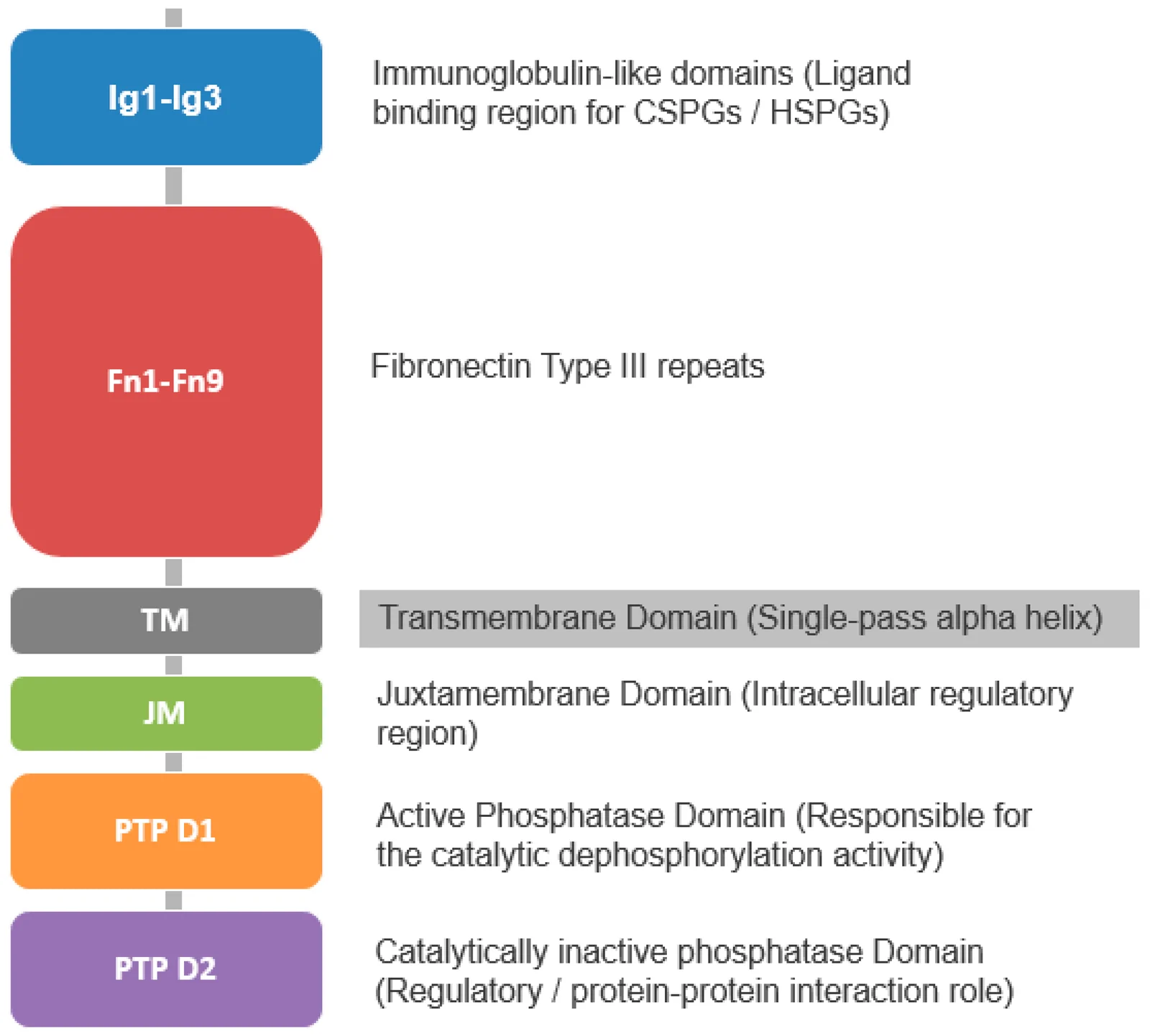
Domain architecture and therapeutic entry points of PTPσ. PTPσ is a type IIA receptor-type protein tyrosine phosphatase composed of three N-terminal extracellular immunoglobulin-like domains (Ig1–Ig3), followed by nine fibronectin type III-like domains (Fn1–Fn9), a single-pass transmembrane (TM) helix, a cytoplasmic juxtamembrane regulatory region, and tandem phosphatase domains D1 and D2. Ig1–Ig2 contains the principal glycosaminoglycan-binding surface responsible for interactions with heparan sulfate- and chondroitin sulfate-bearing proteoglycans. Fn9 is a membrane-proximal extracellular regulatory domain targeted by function-modulating antibodies. D1 is the principal catalytically active domain, whereas D2 is a catalytically impaired PTP-like regulatory domain. The scheme also indicates representative therapeutic entry points: ligand-intercepting Ig1–Ig2 decoys, Fn9-directed antibodies or soluble Fn9 decoys, intracellular wedge peptides, and catalytic/allosteric inhibitors.

**Figure 2:**
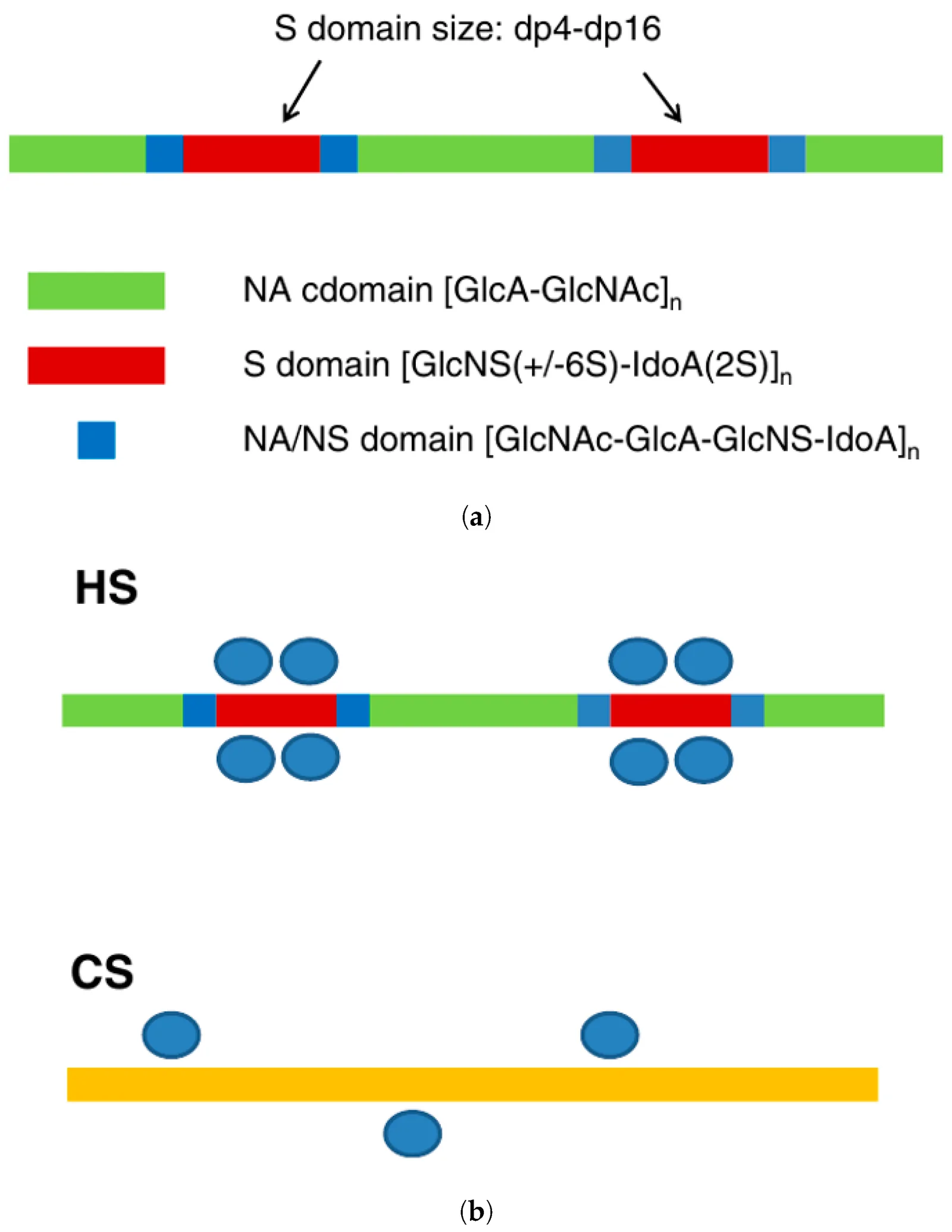
Differential regulation mechanisms of PTPσ activity by proteoglycans. (**a**) Domain structure of HSPG. In heparan sulfate proteoglycan (HSPG), there are islands (S domain) of highly sulfated regions of dp4–16 (red). NAc, N-acetylated; GlcA-GlcNAc, (glucuronic acid)-(N-acetylated glucosamine); GluNS(+/−6S)-IdoA(2S), (N-sulfoglucosamine (+/−6S))-(Iduronic acid(2S)); NA/NS, N-acetylated/N-sulfated. (**b**) PTPσ regulation by different domain structures of heparan sulfate (HS) and chondroitin sulfate (CS). The proteoglycan-binding PTPσ Ig1–Ig2 domains are represented as blue circles (one circle for one Ig1–Ig2) on the strips of proteoglycans HS and CS. The highly sulfated islands (red) in HS mediate an efficient oligomerization of PTPσ. The oligomerization of PTPσ molecules can generate uneven distribution of PTP activity either by dimerization-mediated enzyme inhibition or depletion of enzyme in the regions lacking oligomerization. The CS with even distribution of medium- or low-sulfated disaccharide units cannot mediate efficient oligomerization of PTPσ. Figure adapted from Chien and Ryu [19] under the Springer Nature License Terms and Conditions.

**Figure 3:**
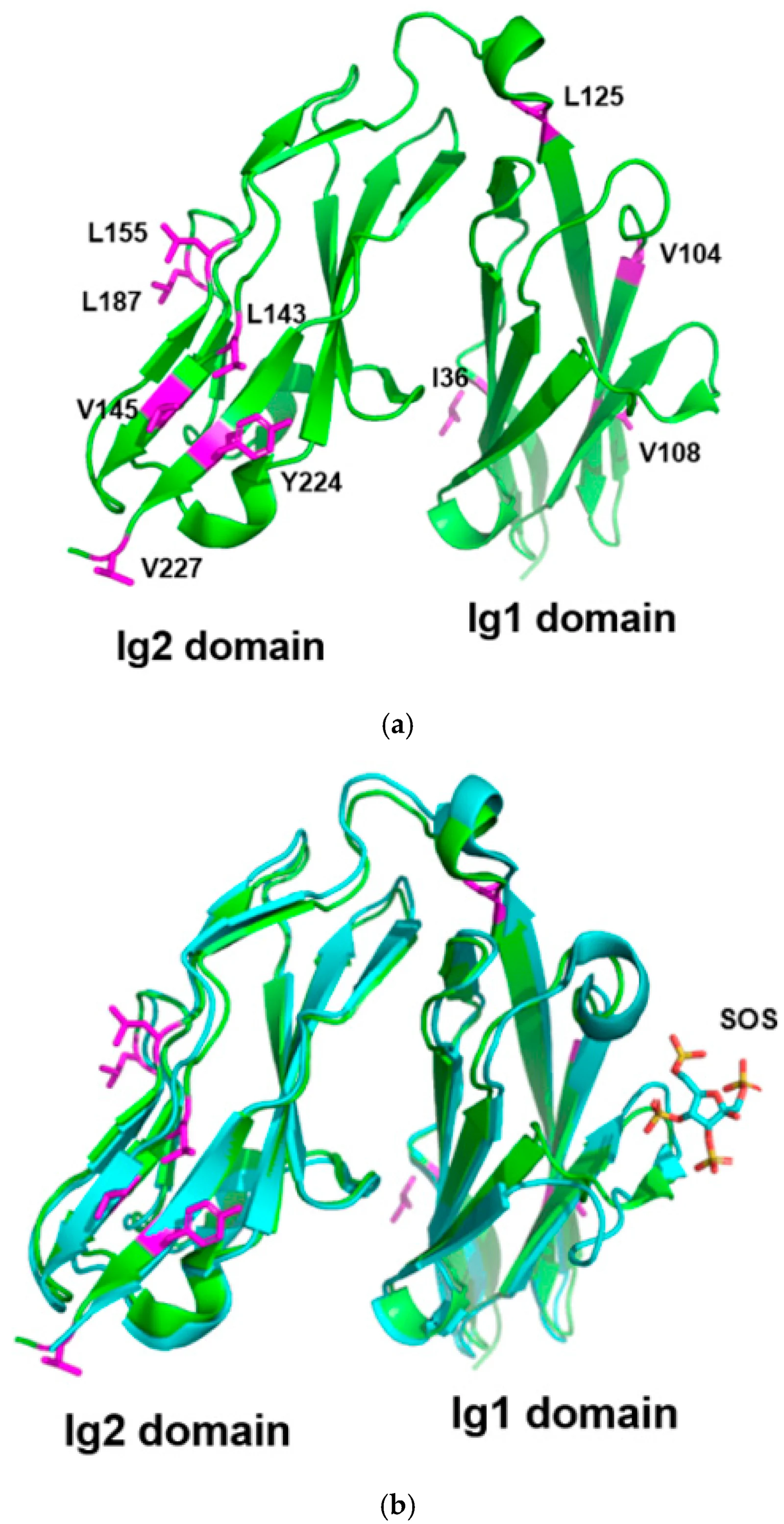
Structure-guided engineering of the PTPσ Ig1–Ig2 decoy. (**a**) Human PTPσ Ig1–Ig2 crystal structure (PDB: 2YD2) [17] showing solvent-exposed hydrophobic residues selected for mutagenesis. The engineered residues L143, V145, L155, and L187 are spatially separated from the principal sulfated-glycan-binding surface, providing a rationale for improving solubility while preserving ligand recognition. Side chains of the structure-based mutation sites are represented on the figure. (**b**) Structural comparison of human PTPσ Ig1–Ig2 with the sulfated-glycan binding mode observed in the homologous LAR/PTPRF Ig1–Ig2–sucrose octasulfate complex (PDB: 2YD8) [17]. Sucrose octasulfate (SOS) is used as a chondroitin sulfate/heparan sulfate mimetic. This comparison illustrates that the engineered surface residues lie outside the conserved basic glycosaminoglycan-binding region. Figure reproduced from Park et al. [33] under the CC BY 4.0 license.

**Figure 4:**
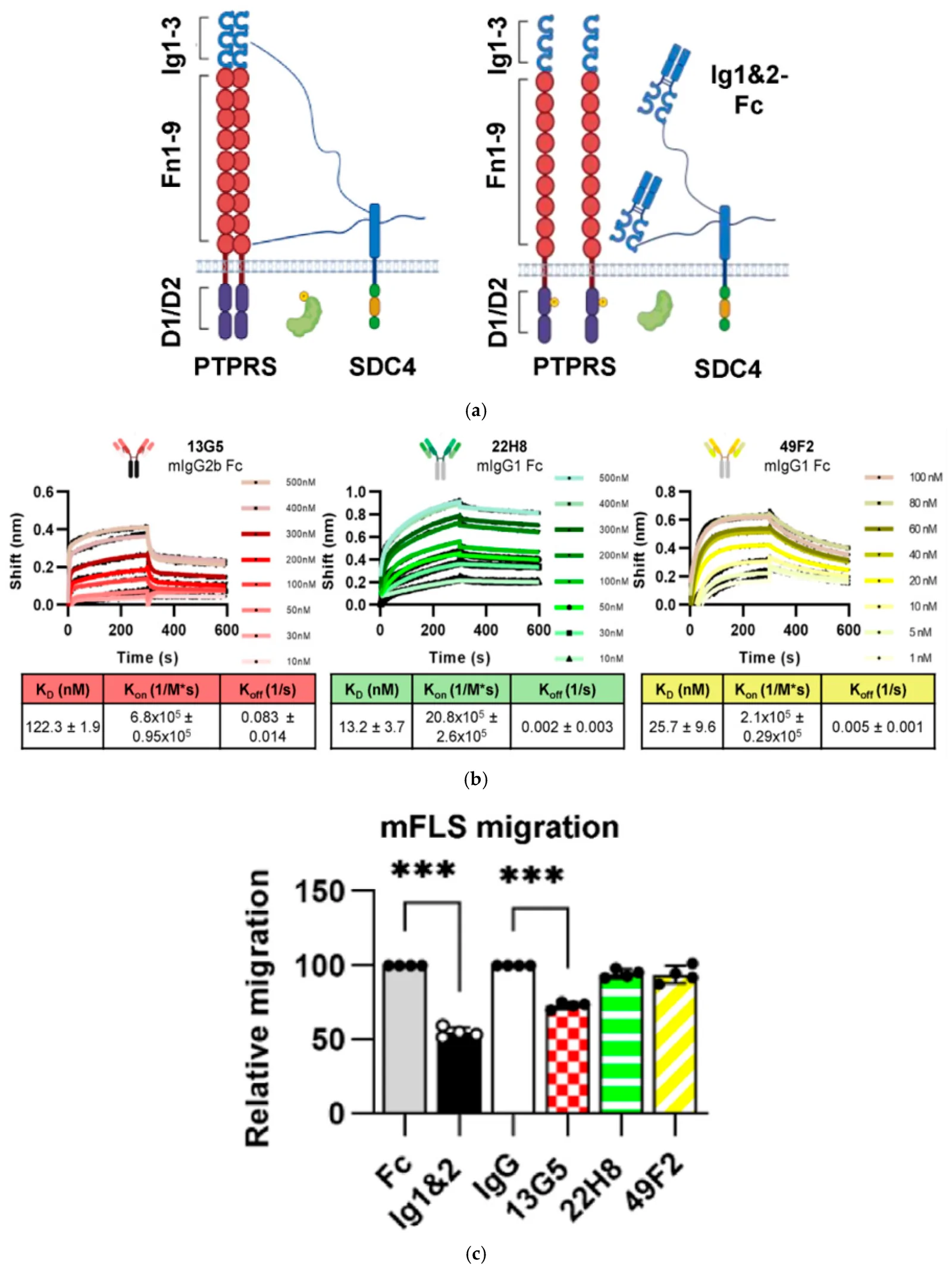
Extracellular PTPσ-targeting formats can alter PTPσ–syndecan-4 association, receptor oligomerization, and fibroblast-like synoviocyte migration. (**a**) Scheme of the proposed mechanism by which soluble PTPσ Ig1–Ig2–Fc competes for proteoglycan-associated ligands and thereby reduces productive PTPσ interaction with syndecan-4 (SDC4). (**b**) Biolayer interferometry sensorgrams for antibodies 13G5, 22H8, and 49F2 interacting with the PTPσ extracellular domain. Concentration ranges and fitted kinetic parameters are indicated as reported in the original study. (**c**) Relative transwell migration of mouse fibroblast-like synoviocytes after treatment with Fn9-directed antiPTPσ antibodies or PTPσ Ig1–Ig2–human Fc decoy. Human Fc and mouse IgG are negative controls. Each point represents one biological replicate; statistical analysis and replicate number are reported as in the original publication. The figure illustrates that similar binding-site assignment or affinity does not necessarily predict equivalent cellular activity, consistent with a role for epitope geometry and receptor-state control. Figure reproduced from Hein Da Rosa et al. [31] under the CC BY 4.0 license. *** *p* ≤ 0.001.

**Table 1:** Characteristics of PTP subclasses and therapeutic relevance.

Feature	Classical PTPs	Type IIA RPTPs (LAR/PTPδ/PTPσ)	PTPσ-Specific Therapeutic Relevance
Therapeutic target region	Intracellular catalytic site	Extracellular domains plus intracellular catalytic site	Extracellular targeting enables antibody and decoy strategies
Ligand biology	Often protein interactions and intracellular complexes	Cell-surface/ECM ligands, adhesion complexes, proteoglycans	Proteoglycan switch links HS/CS presentation to receptor regulation
Catalytic regulation	Active-site and allosteric regulation	Ligand-dependent receptor association plus D1/D2 regulation	Disease-specific activation versus inhibition can be engineered
Major druggability issue	Conserved, polar active site; cell permeability	Epitope geometry, clustering, avidity, and delivery	Ig1–Ig2 and Fn9 provide differentiated extracellular entry points

**Table 2:** Status of PTPσ structural data.

Domain	Experimental Structure	PDP Code	Potential Usage
Ig1–Ig2	Yes	2YD1–2YD9 (representative PDB: 2YD2) [17]	CSPG/HSPG binding region, soluble decoy engineering
N-terminal 6 domains	Yes	4PBX [32]	Relative orientation and flexibility of domains in receptor regulation *
TrkC-binding domain (Ig1–Ig3)	Yes	4PBW [32]	Trans-synaptic TrkC–PTPσ interface
Cytoplasmic D1–D2	Yes	2FH7 [39], 3SR9 [40], and 4BPC [37]	Catalytic pocket, D1–D2 interface, allosteric inhibitor design
Ig3–Fn9	No	No	Relative orientation and flexibility of domains in receptor regulation *
Fn9	No	No	Anti-Fn9 antibody epitope mapping
Full-length receptor including TM helix	No	No	Membrane context, dimer/oligomer state analysis

* The elongated multi-domain arrangement of PTPσ ECDs may contribute to the fine regulation of receptor activity.

**Table 3:** Comparative summary of PTPσ-targeting therapeutic modalities.

Modality/Representative Format	Primary Target Site	Mechanism of PTPσ Modulation	Intended Direction of PTPσ Modulation	Disease Context and Therapeutic Rationale	Key Development Considerations
Soluble Ig1–Ig2 decoy (Ig1–Ig2-Fc; engineered variants) [14,21,33,35].	Extracellular CSPG/HS ligands.	Reduces productive ligand engagement of cell-surface PTPσ. Fc fusion can increase avidity and half-life.	Decreases CSPG-driven PTPσ inhibitory signaling in neural injury; disruption of HSPG-mediated regulation for PTPσ reactivation in FLS.	CNS injury, spinal cord/peripheral nerve injury, stroke: reduces CSPG–PTPσ growth-inhibitory signaling. RA: interferes with HSPG-dependent PTPσ suppression and restrains pathogenic FLS motility.	Native fragment has poor solubility; requires developability engineering while retaining glycan binding. CNS programs require lesion/CNS delivery strategy; RA can consider systemic or intra-articular dosing.
Fn9-directed antibody (13G5 IgG1; scFv-Fc) [31].	Membrane-proximal Fn9 ectodomain.	Alters Fn9-dependent receptor association.	Restores/increases PTPσ activity in FLS.	RA: reactivated PTPσ suppresses FLS migration and invasion; engineered 13G5 IgG1 reduced clinical and histological arthritis measures in a mouse model.	Functional activity is not predicted by affinity alone. Epitope, Fc isotype, hinge geometry and valency may determine receptor-state pharmacology.
Soluble Fn9 domain decoy (Fn9-Fc) [31].	Fn9-mediated extracellular interactions.	competes with or perturbs Fn9-dependent interactions.	Restores/increases PTPσ activity in FLS.	RA: reduced FLS migration in a PTPσ-dependent manner; an alternative to direct receptor-binding antibodies.	Needs validation of specificity and the precise binding partner(s) and optimization of avidity and stability.
Intracellular wedge peptide (TAT-conjugated ISP) [27,28].	Juxtamembrane “wedge” regulatory region; intracellular face.	Cell-penetrating peptide binds PTPσ and attenuates PTPσ-dependent inhibitory signaling.	Decreases PTPσ-mediated inhibitory signaling/activity.	Spinal cord injury and other neural repair settings; reported to promote axonal crossing, serotonergic innervation and functional recovery in rodent models.	Cell penetration, CNS/lesion biodistribution, protease stability, dose/exposure control and nonspecific CPP effects are central risks. Optimize LAR-family selectivity, formulation and scalable peptide manufacturing.
Small-molecule allosteric inhibitor (DJ001-like) [36].	Intracellular allosteric regulatory site.	Noncompetitive allosteric inhibition of PTPσ, aiming to relieve its negative regulation of HSC regeneration.	Decreases PTPσ activity.	Bone-marrow injury and hematopoietic regeneration after radiation or myelosuppressive chemotherapy.	needs rigorous PTPσ versus LAR-family selectivity, intracellular exposure, off-target pharmacology and chronic-safety assessment.

**Table 4:** Comparison of wild-type and representative engineered variants.

Parameter	Wild Type	Representative Engineered Variant	Interpretation
Production yield	1.0	L155N/L187N: 8.8-fold	Markedly improved in CHO transient expression [33]
Relative solubility	21%	L155N/L187N: 100%	No precipitation loss was observed during concentration [33]
Purification stability	99% pure	L155N/L187N: similar to WT	Strong and 99% pure band was obtained in SDS-PAGE [33]
CS binding	Retained	L155N/L187N: similar to WT	Ligand recognition required for decoy activity is preserved [33]
Neuronal cell assay	Limited at high concentrations	Improved with L155N/L187N	Increased solubility likely improves effective exposure [33]

**Table 5:** Indication areas and development considerations.

Indication Area	Biological Rationale	Candidate Modality
Spinal cord injury, peripheral nerve injury	Increased CSPG at injury sites and PTPσ-mediated inhibition of regeneration [14,15,16,17,18,27,28,29]	CSPG decoy (Ig1–Ig2), local delivery
Neurodegeneration, including Alzheimer’s disease and stroke	Reported links between PTPσ, synaptic integrity, and tau pathology [34]	CSPG-decoy (Ig1–Ig2) or antibody coupled to BBB-shuttle
Rheumatoid arthritis	Synovial fibroblast invasiveness and proteoglycan switching [13,30,31]	HSPG-decoy (Ig1–Ig2), Fn9-antibody in combination with a TNF inhibitor
Bone marrow injury and hematopoietic regeneration	Preclinical evidence that PTPσ inhibition promotes HSC regeneration [36]	Selective small molecule/peptide or biologic

## Data Availability

No new data were created or analyzed in this study. Data sharing is not applicable to this article.

## Abbreviations

AD, Alzheimer’s disease; BBB, blood–brain barrier; CNS, central nervous system; CPP, cell-penetrating peptide; CS, chondroitin sulfate; CSPG, chondroitin sulfate proteoglycan; D1, membrane-proximal phosphatase domain 1; D2, membrane-distal phosphatase domain 2; ECD, extracellular domain; FLS, fibroblast-like synoviocyte; Fn, fibronectin type III-like domain; GAG, glycosaminoglycan; HSC, hematopoietic stem cell; HS, heparan sulfate; HSPG, heparan sulfate proteoglycan; Ig, immunoglobulin-like domain; ISP, intracellular sigma peptide; LAR, leukocyte common antigen-related phosphatase; NgR, Nogo receptor; PDB, Protein Data Bank; PTP, protein tyrosine phosphatase; PTPRS, protein tyrosine phosphatase receptor type S gene; PTPσ, receptor-type tyrosine-protein phosphatase sigma; RA, rheumatoid arthritis; RPTP, receptor-type protein tyrosine phosphatase; SDC4, syndecan-4; SOS, sucrose octasulfate; TM, transmembrane.
